# Six splice site variations, three of them novel, in the ABO gene occurring in nine individuals with ABO subtypes

**DOI:** 10.1186/s12967-021-03141-5

**Published:** 2021-11-22

**Authors:** Xiaozhen Hong, Yanling Ying, Jingjing Zhang, Shu Chen, Xianguo Xu, Ji He, Faming Zhu

**Affiliations:** 1 Blood Center of Zhejiang Province, Jianye Road 789, Hangzhou, Zhejiang 30052 People’s Republic of China; 2 Key Laboratory of Blood Safety Research of Zhejiang Province, Hangzhou, Zhejiang 310052 People’s Republic of China

**Keywords:** ABO subtype, Splice site, Variation, Polymerase chain reaction sequence-based typing

## Abstract

**Background:**

Nucleotide mutations in the *ABO* gene may reduce the activity of glycosyltransferase, resulting in lower levels of A or B antigen expression in red blood cells. Six known splice sites have been identified according to the database of red cell immunogenetics and the blood group terminology of the International Society of Blood Transfusion. Here, we describe six distinct splice site variants in individuals with ABO subtypes.

**Methods:**

The ABO phenotype was examined using a conventional serological method. A polymerase chain reaction sequence-based typing method was used to examine the whole coding sequence of the *ABO* gene. The *ABO* gene haplotypes were studied using allele-specific primer amplification or cloning technology. In silico analytic tools were used to assess the functional effect of splice site variations.

**Results:**

Six distinct variants in the *ABO* gene splice sites were identified in nine individuals with ABO subtypes, including c.28 + 1_2delGT, c.28 + 5G > A, c.28 + 5G > C, c.155 + 5G > A, c.204-1G > A and c.374 + 5G > A. c.28 + 1_2delGT was detected in an A_w_ individual, while c.28 + 5G > A, c.28 + 5G > C, and c.204-1G > A were detected in B_el_ individuals. c.155 + 5G > A was detected in one B_3_ and two AB_3_ individuals, whereas c.374 + 5G > A was identified in two A_el_ individuals. Three novel splice site variants (c.28 + 1_2delGT, c.28 + 5G > A and c.28 + 5G > C) in the *ABO* gene were discovered, all of which resulted in low antigen expression. In silico analysis revealed that all variants had the potential to alter splice transcripts.

**Conclusions:**

Three novel splice site variations in the *ABO* gene were identified in Chinese individuals, resulting in decreased A or B antigen expression and the formation of ABO subtypes.

**Supplementary Information:**

The online version contains supplementary material available at 10.1186/s12967-021-03141-5.

## Background

Karl Landsteiner discovered the ABO blood group system in 1900, and it was of great clinical significance in blood transfusion and organ transplantation [1]; additionally, it is important for studying the development of numerous human diseases [2–4]. Incompatibility in the ABO blood group may result in severe haemolytic reactions during transfusion and neonatal haemolytic disease [5, 6].

The *ABO* gene is located on chromosome 9; its full-length sequence is approximately 24.9 kb, and the coding region is organized into seven exons with 28, 70, 57, 48, 36, 135, and 691 nucleotide base pairs [7, 8]. Five *ABO* blood group alleles, including ABO*A1.01, ABO*A1.02, ABO*B.01, ABO*O.01.01, and ABO*O.01.02, are common in the Chinese Han population [9]. The *ABO* gene encodes glycosyltransferase A (GTA) or glycosyltransferase B (GTB), which catalyse the formation of A or B antigens on red blood cells, respectively [8]. However, there are only seven nucleotide changes between the ABO*A1.01 and ABO*B.01 alleles in the coding sequence (CDS) region, resulting in four amino acid alterations between GTA and GTB [8, 10].

Although the ABO blood group system is composed of four phenotypes, including A, B, O, and AB, the distribution of ABO phenotypes varies across populations and regions [9]. Additionally, some subtypes of ABO phenotypes have been identified in populations, which often exhibit differences in forward and reverse ABO typing due to reduced antigen and/or antibody expression [9, 11, 12]. Variations in the *ABO* gene may affect the activity and/or specificity of glycosyltransferases, resulting in the formation of ABO subtypes. According to the database of names for ABO blood group alleles (v1.1) by the red cell immunogenetics and blood group terminology of international society of Blood Transfusion (ISBT), five splice sites with Aweak or Ael and one splice site with B3 were found in populations from around the world. In this study, we described six different splice site variants of the *AB*O gene in individuals with ABO subtypes.

## Materials and methods

### Study specimens

Individuals with ABO subtypes were either blood donors or patients. All specimens were obtained after the individuals provided informed consent. This study was approved by the ethics committee of the Blood Center of Zhejiang Province, China. The difference in these specimens was discovered during routine ABO blood group typing. The specimens placed in tubes with or without EDTA anticoagulant were sent to the immunohaematology reference laboratory in the Blood Center of Zhejiang Province for further analysis.

### Serological tests

A, B, and H antigens, as well as anti-A and anti-B antibodies, were detected using a conventional serological method [13, 14]. Anti-A, anti-A_1_, anti-B, anti-AB, and anti-H antibody reagents were used (Shanghai Blood Biotechnology Co., Ltd., Shanghai, China). Red blood cells (RBCs) in groups A, B, and O were prepared in the laboratory using fresh blood from three donors of the same type at random.

### *ABO* gene full exon sequencing analysis

According to our previous reports, we used the polymerase chain reaction sequence-based typing (PCR-SBT) technique to analyse the entire CDS of the *ABO* gene [9, 13, 14]. Three sets of primers were used to amplify all exons of the *ABO* gene. The amplicons were purified, followed by sequencing and analysis using an ABI 3730 sequencer (Applied Biosystems, Foster City, CA, USA). SeqScape v2.5 software (Applied Biosystems) was used to evaluate the sequencing data. The *ABO* gene reference sequence was obtained from GenBank (ID number NG_006669.2), and the *ABO* genotype was assigned based on nucleotide polymorphism. The *ABO* allele was nominated in accordance with the ISBT guidelines for red cell immunogenetics and blood group terminology [15].

### *ABO* gene sequence analysis using NGS

Sequences from the start codon to the stop codon of the *ABO* gene were analysed using next-generation sequencing (NGS). First, the *ABO* gene sequence was amplified using two pairs of primers. In the first pair, the forward and reverse primer sequences were 5’GCGCCGTCCCTTCCTAGCAG 3’ and 5’AGCCACCAACTTCCCCTAGT3’. The primer sequences in the second pair were 5’TACTCACCTATTATTGGCCTTTGGTT3’ and 5’TAGGCTTCAGTTACTCACAACAGGAC3’. The expected lengths of the amplicons were approximately 12,763 and 7250 bp, respectively. The total volume for each PCR amplification reaction was 25 μL, which included 5 × GLX PCR buffer 5 μL (Takara Bio Company, Dalian, China), 200 μmol/L dNTP concentration, 0.2 μmol/L primer concentration, 0.625 U GLX Taq enzyme (Takara Bio Company) and 2.5 μL DNA sample. Amplification was performed on an ABI PCR 9700 instrument (Applied Biosystems). The following conditions were used for PCR amplification: predenaturation at 94 ℃ for 1 min, denaturation at 98℃ for 10 s, annealing at 68℃ for 10 min, 30 cycles, and extension at 68℃ for 10 min. The amplicons were digested with Tn5 transposase, and the index was added to construct the library using the Trans NGS Tn5 DNA library prep kit for Illumina (Transgene, Beijing, China). All procedures were carried out strictly according to the manufacturer’s instructions. Following the qualification of the library's quality, the sequences were detected on an Illumina MiSeq Sequencer using the MiSeq sequencing reagent kit (V2, 300 cycles, Illumina Inc., San Diego, CA, USA). The sequencing data were analysed using the *ABO* reference sequence (GenBank ID number NG_006669.2 for genomic, NM_020469.2 for transcript) and CLC main workbench 12.0 software (Qiagen company, Hilden, Germany), and all polymorphism nucleotides were recorded and analysed.

### Analysis of the *ABO* gene haplotype

Allele-specific primer amplification sequencing or cloning technology was used to haplotype the *ABO* gene [9, 13]. For allele-specific primer amplification (specimen ID numbers 4 to 9), specific primers for the *A, B,* and *O* alleles were used to amplify the corresponding alleles, and the amplicons were then sequenced and analysed as previously reported [9, 13]. For cloning technology (specimen ID numbers 1 to 3), the PCR-SBT amplicon was ligated with the pCR4^@^TOPO plasmid vector according to the manufacturer’s instructions and transfected into competent cells to grow. As previously described, plasmid DNA was extracted as a template for sequencing analysis [9, 13].

### In silico splicing transcript analysis

Alamut® software v2.10 was used in conjunction with four splice site prediction tools, SpliceSite Finder-like, MaxEntScan, NNSPLICE, and GeneSplicer, to predict the effects of these splice site variations (www.interactive-biosoftware.com/doc/alamut-visual/2.6/splicing.html) [16–18]. The Berkeley Drosophila Genome Project Searches Splice Site Prediction software and NetGene2-2.42 software were also used to predict potential splicing transcripts [19, 20]. A splice site score calculator was used to assess the strength of the constitutive and cryptic acceptor splicing sites. A ≥ 10% change in the splice site signal in at least two algorithms was considered to have an effect on splicing [21].

## Results

### The ABO subtypes' phenotypes

Nine Chinese individuals with ABO blood group typing inconsistencies were studied. Four were blood donors, while the rest were patients. Table 1 shows the agglutination reaction states of these individuals’ RBCs with anti-A, anti-A_1_, anti-B, anti-AB, anti-H, and serum containing known A, B, O group RBCs. All individual RBCs exhibited 3 + or 4 + strength agglutination with anti-H. In the absorption and elution test, B antigen was expressed in ID numbers 2, 3, and 7 specimens, whereas A antigen was positive in ID numbers 8 and 9 specimens. Individuals with ID numbers 4, 5, and 6 had mixed-field agglutination. These individuals were classified as ABO subtypes based on serological characteristics (Table 1), with 3 individuals belonging to subtype A and 6 individuals belonging to subtype B.

**Table 1:** Serological and genotype results in the samples with ABO subtypes

ID	Phenotype	Genotype#	Forward typing					Reverse typing		
			Anti-A	Anti-B	Anti-AB	Anti-A1	Anti-H	Ac	Bc	Oc
1	A_w_	ABO*A1.02/ABO*O.01.01 with c.28. + 1_-_2delGT	1 +	0	1 +	0	4 +	1 +	4 +	0
2	B_el_	ABO*B.01/ABO*O.01.02 with c.28 + 5G > A	0	0	0	0	4 +	4 +	0	0
3	B_el_	ABO*B.01/ABO*O.01.02 with c.28 + 5G > C	0	0	0	0	4 +	4 +	0	0
4	B_3_	ABO*B3.03/ABO*O.01.01	0	mf	2 +	0	3 +	2 +	0	0
5	AB_3_	ABO*A1.02/ABO*B3.03	4 +	1 + mf	4 +	4 +	3 +	0	0	0
6	AB_3_	ABO*A1.02/ABO*B3.03	4 +	3 + mf	4 +	3 +	4 +	0	0	0
7	B_el_	ABO*B.01/ABO*O.01.02 with c.204-1G > A	0	0	0	0	4 +	4 +	1 +	0
8	A_el_	ABO*AEL.new/ABO*O.01.02	0	0	0	0	4 +	±	3 +	0
9	A_el_	ABO*AEL.new/ABO*O.01.01	0	0	0	0	4 +	1 +	4 +	0

^#^ABO*B3.03 was ABO*B.01 with c.155 + 5G > A; ABO*AEL.new was ABO*A1.02 with c.374 + 5G > A. *Ac* A cells, *Bc* B cells, *Oc* O cells. 1 + to 4 +  = agglutination of increasing strength; mf = mixed-field agglutination; 0 = no agglutination. Nucleotide position 1 is identical to the first nucleotide of the coding sequence

### Analysis of the *ABO* gene's CDS region

All nucleotides for full exons of the *ABO* gene in nine individuals were sequenced, but no variation was observed in the CDS regions. Table 1 shows the *ABO* genotypes of the individuals based on the sequences of all exons. Further sequence analysis of exon and intron splicing acceptor/donor sites in the nine individuals showed six distinct heterozygotes at positions c.28 + 1_2, c.28 + 5, c.155 + 5, c.204–1, and c.374 + 5 (Additional file 1: Figure S1).

### *ABO* gene sequence analysis using NGS

Additional file 2: Table S1 lists 166 polymorphic nucleotides of these ABO subtypes (ID numbers 3 to 8) compared to *ABO* gene sequences in the GenBank database (NG_006669.2 for genomic, NM_020469.2 for transcript) (sequence between start codon and stop codon in the *ABO* gene). The ID numbers 1, 2, and 9 specimens were not analysed using the NGS method. Except for the splicing acceptor/donor sites, which were consistent with the PCR-SBT, no variation was observed in the NGS method.

### The *ABO* gene's haplotype

The haplotyping analysis revealed six distinct splice site variants in the ABO subtypes, including c.28 + 1_2delGT, c.28 + 5G > A, c.28 + 5G > C, c.155 + 5G > A, c.204-1G > A, and c.374 + 5G > A (Additional file 1: Figure S2). c.155 + 5G > A was identified in three ABO subtype individuals, c.374 + 5G > A in two individuals, and c.28 + 1_2delGT, c.28 + 5G > A, c.28 + 5G > C, c.204-1G > A variants in one individual. The sequences for all variants were submitted to the GenBank Database, with the nucleotide sequence and accession numbers listed in Table 2.

**Table 2:** The splice probability of the variant types in silico analysis using different tools

Variants	ISBT Allele name	GenBank ID number	gnomAD v2.1.1 ID number#	dbSNP ID number	Frequency	Nearest Type	SSF (0–100)			MaxEnte(0–16)			NNS(0–1)			GS(0–15)			Splice probability	
							Score		Change	Score		Change	Score		Change	Score		Change		
							WT	MUT	%	WT	MUT	%	WT	MUT	%	WT	MUT	%		
c.28. + 1_2delGT	Novel	MK393880	g.136150576_77delCA	rs5442107549	NA	5'	86.72	0	100	10.9	0	100	0.99	0	100	15.11	0	100	1	
c.28 + 5G > A	Novel	MT877223	g.136150573C > T	rs5442107547	NA	5'	86.72	74.57	14.01	10.9	4.89	55.14	0.99	0.28	71.72	15.11	8.84	41.5	0.996	
c.28 + 5G > C	Novel	MN540965	g.136150573C > G	rs5442107548	NA	5'	86.72	74.03	14.63	10.9	4.54	58.35	0.99	0.16	83.84	15.11	7.91	47.65	0.997	
c.155 + 5G > A	ABO*B3.03	MN540966	g.136136716C > T	rs782187929	0.0009%	5'	87.13	74.98	13.94	10.1	3.53	65.05	0.97	0.19	80.41	9.02	2.59	71.29	0.998	
c.204-1G > A	Novel	MN540964	g.136133523C > A	rs1444418339	0.0008%	3'	80.97	0	100	7.45	0	100	0.44	0	100	5.36	0	100	1	
c.374 + 5G > A	ABO*AEL.04	MN540967	g.136132791C > T	rs1289213676	0.0004%	5'	84.81	72.66	14.33	9.82	3.59	63.44	0.99	0.72	27.27	3.14	0	100	0.998	

^*^SSF SpliceSiteFinder-like, *MaxEnte* MaxEntScan, *NNS* NNSPLICE, *GS* GeneSplicer, *WT* wild type, *MUT* mutation

*NA* not applicable. #The position in gnomAD v2.1.1 is referenced from NC_000009.11 in gnomAD v2.1.1. Value in parentheses of the tools is score range. Nucleotide position 1 in the variants is identical to the first nucleotide of the coding sequence

Searches for these variants identified in the subtypes in the gnomAD v2.1.1 and dbSNP databases revealed that c.155 + 5G > A (NC_000009.11:g.136136716C > T, GRCh37), c.204-1G > A (NC_000009.11:g.136133523C > A) and c.374 + 5G > A (NC_000009.11:g.136132791C > T) existed in these databases with frequencies of 0.0009%, 0.0008%, and 0.0004%, respectively. However, c.28 + 1_2delGT (NC_000009.11: g.136150576_77delCA), c.28 + 5G > A (NC_000009.11: g.136150573C > T), and c.28 + 5G > C (NC_000009.11: g.136150573C > G) were identified for the first time in the ABO subtypes (Table 2).

### In silico predictions to assess the functional implications of splice site variations

Table 2 shows the changes in the splice site signal of the variants using the SpliceSite Finder-like, MaxEntScan, NNSPLICE, and GeneSplicer tools. All variants had a probability of affecting the splice transcripts (probability over 0.99). The Berkeley Drosophila Genome Project Searches Splice Site Prediction software identified over 40 new donor sites in the c.28 + 1_2delGT, c.28 + 5G > C, and c.28 + 5G > A variants. This program identified two donor sites closest to exon 1 at positions c.28 + 168 (cgggcag**GT**gggctc) and c.28 + 267 (ggtcctg**GT**gagagc), with scores of 0.40 and 0.93, respectively. NetGene2-2.42 software predicted c.28 + 267 (ggtcctg**GT**gagagc) as a donor site but not c.28 + 168.

In the in silico analysis using the Berkeley Drosophila Genome Project Searches Splice Site Prediction software, several new splice sites were predicted in the c.155 + 5G > A, c.204-1G > A, and c.374 + 5G > A variants. Some new donor sites were predicted at c.155 + 507 (acataag**GT**aggagg) with a score of 0.95 and c.374 + 840 (ctcctta**GT**aagagg) with a score of 0.51. One of the new acceptor sites was predicted to be located at position c.204–224 (ctcttgcc**AG**tttgtaag) with a score of 0.84. However, some additional spliceosomes resulting from variations in the splice sites might generate partial functional transferases as a result of the RBCs of the probands.

## Discussion

The common ABO subtypes are A_3_, A_x_, A_el_, B_3_, B_x_, B_el_, B_m_, B(A), cisAB, etc. Seltsam A et al. reported that the B_W_ phenotype is caused by variations in the CCAAT-binding factor/NF-Y enhancer region of the *ABO* gene [22]. Sano R demonstrated for the first time that deletion of the *ABO* gene's erythroid cell-specific regulatory element could downregulate transcription in the *B(m)* allele [23]. Numerous *ABO* variations have been identified in individuals with ABO subtypes to date [24–29]. These variants of the *ABO* gene are located in the CDS region, intron 1 erythroid-specific regulatory element region, splice site, promoter, cis- or trans-regulatory element, etc. Kronstein-Wiedemann R et al. found that miR-331-3p and miR-1908-5p directly target the mRNA of GTA and GTB and that overexpression of these miRNAs in haematopoietic stem cells may result in a significant reduction in the expression of A antigens [30]. Some variations in the splice sites of the *ABO* gene are associated with some ABO subtypes. Chen DP et al. reported c.155 + 5G > A (IVS3 + 5G > A) in a B3 individual and c.374 + 5G > A (IVS6 + 5G > A) in an Ael individual^31^, ^32^. In theory, changes in the *ABO* gene splice site result in the formation of new RNA splice sites and therefore novel versions of ABO mRNA.

The Chinese population has a high prevalence of ABO subtypes [13, 14]. In our research, we routinely analysed the *ABO* gene's full CDS and the sequence of the erythroid cell-specific regulatory element region for ABO subtypes using the PCR-SBT technique. We discovered over 50 novel alleles from ABO subtypes [9, 13, 33]. In this study, six distinct splicing site variants in the *ABO* gene were identified in nine individuals with ABO subtypes. Between 2015 and 2019, our laboratory screened and obtained specimens from 369 individuals with suspected ABO subtypes using a combination of serological and molecular methods.

Multiple ABO mRNA forms were detected in the normal ABO phenotype by RT–PCR, the majority of which lacked exon 6 [34, 35]. However, in some individuals with ABO subtypes, RNA splicing of the *ABO* gene was detected [31, 32, 36, 37]. An ABO* A1-like allele with a 4 bp deletion (c.236-239delCGTG) in exon 5 and a 20 bp downstream deletion in intron 5 affected the donor splice site [36]. c.28G > A in exon 1 is associated with the weak B subtype via its effect on the *ABO* gene's RNA splicing [37]. Previously, c.155 + 5G > A was discovered in B3 individuals [31]. At least 7 distinct types of splicing transcripts were identified in B3 individuals [31]. While it is possible to generate a mRNA without the matching exon 3 fragment, only one of 102 mRNA clones contained an exon 3 deletion splicing variant, suggesting that further variable splicing occurred [31]. The c.374 + 5G > A variant originally identified on the ABO*A1.01 allele in the Ael individual, currently referred to as the ABO*AEL.04 allele*,* is predicted to generate transcripts without exon 6 or exons 5 to 6^32^. At least 10 distinct splicing transcript types were identified in the ABO*AEL.04 individual [31]. However, this study discovered a c.374 + 5G > A variant in the ABO*A1.02 allele.

Hwang DY et al. found that the IVS1 + 2 T > C in intron 1 of the *CYP17A1* gene could result in cryptic splicing, with the splicing transcripts being included in exon 1 [38]. In this study, the c.28 + 1_2delGT, c.28 + 5G > A, and c.28 + 5G > C variants of the *ABO* gene were found to be located in the exon/intron 1 boundary. We hypothesized that these variations would impair adjacent intron splicing and induce alternate activation of some cryptic splice donor sites within exon 1, resulting in aberrant mRNA splicing. One of the predicted splice sites closest to exon 1 can produce a protein with an additional 89 amino acids. Additionally, Kominato Y et al. found alternative exon 1a in the upstream genomic sequence of the *ABO* gene [34]. As a result, alternative splicing transcripts may begin with exon 1a in individuals with the c.28 + 1_2delGT, c.28 + 5G > A, and c.28 + 5G > C variants. This possibility needs to be further confirmed in subsequent research.

Alternative 3′ or 5′ splice sites have been shown to be capable of skipping exons in the transcripts [39]. The c.155 + 5G > A, c.204-1G > A, and c.374 + 5G > A variations should generate new transcripts skipping exons 3, 5, and 6, respectively, in the in silico prediction. Because of a lack of the corresponding exon sequence or the formation of new spliceosomes, the amino acid sequence of the glycosyltransferase varies, affecting the catalytic activity. Splicing transcripts lacking exon 3 or exon 5 are predicted to generate functional glycosyltransferases lacking 19 or 12 amino acids at the N-terminus, respectively, whereas splicing transcripts lacking exon 6 could lead to a premature stop codon and form a new premature glycosyltransferase with only 79 amino acid residues. According to the serological findings for the probands, certain alternative spliceosomes would generate functional transferases as a consequence of A or B antigen expression in the RBCs of the probands.

In this study, we predicted splicing transcripts for splice site variations in silico; however, various prediction results for splicing transcripts were discovered using different methods. The scores for the c.155 + 5G > A variant were 0.97 to 0.19 in the NNSPLICE tool and 9.02 to 2.59 in the GeneSplicer tool, and the change ratios were different. Therefore, multiple tools should be used in tandem to predict functional variations. In the normal phenotype, multiple *ABO* mRNA forms may be detected in peripheral blood leukocytes [35]. However, due to a lack of fresh blood samples, *ABO* mRNAs in individuals with these variations were not analysed, and their function in vitro was not examined in our study. Further research is needed in the future to determine the actual status of *ABO* mRNA transcription in the presence of splice site variants.

## Conclusions

In this study, we identified six distinct *ABO* gene splice site variants in individuals with ABO subtypes, including three novel variants, c.28 + 1_2delGT, c.28 + 5G > A and c.28 + 5G > C. Additionally, in silico analysis was used to estimate the potential splicing transcripts for the variants in splice sites. We found that splice site variations in the *ABO* gene affect splice transcripts, resulting in decreased A or B antigen expression and the formation of the ABO subtype.

## Supplementary Information

**Additional file 1: Figure S1**. The heterozygous sequence of the splicing sites in the individuals with ABO subtypes. Arrows indicated the heterozygous variations in ABO gene. **Figure S2**. The variation sequences of the splicing sites after haploid analysis in the ABO gene. Arrows indicated the variation position.

**Additional file 1: Table S1**. Nucleotide polymorphism of the ABO gene in specimens ID number3 to 8 by the NGS method.

## Data Availability

The datasets used and/or analysed during the present study are available upon reasonable request from the corresponding author.

## Abbreviations

GTA: Glycosyltransferase A
GTB: Glycosyltransferase B
PCR-SBT: Polymerase chain reaction sequence-based typing
RBCs: Red blood cells

## References

[1] Landsteiner K (1900). Zur Kenntnis der antifermentativen, lytischen und agglutinierenden. Wirkungen des Blutserums und der Lymphe. Zentralbl Bakteriol.

[2] Franchini M, Liumbruno GM (2013). ABO blood group: old dogma, new perspectives. Clin Chem Lab Med.

[3] Tekgündüz SA, Özbek N (2016). ABO blood group mismatched hematopoietic stem cell transplantation. Transfus Apher Sci.

[4] Franchini M, Mannucci PM (2014). ABO blood group and thrombotic vascular disease. Thromb Haemost.

[5] Tormey CA, Hendrickson JE (2019). Transfusion-related red blood cell alloantibodies: induction and consequences. Blood.

[6] Bel Hadj I, Boukhris R, Khalsi F, Namouchi M, Bougmiza I, Tinsa F (2019). ABO hemolytic disease of newborn: does newborn's blood group a risk factor?. Tunis Med.

[7] Yamamoto F, Clausen H, White T, Marken J, Hakomori S (1990). Molecular genetic basis of the histo-blood group ABO system. Nature.

[8] Yamamoto F (1995). Molecular genetics of the ABO histo-blood group system. Vox Sang.

[9] Zhu F, Tao S, Xu X, Ying Y, Hong X, Zhu H (2010). Distribution of ABO blood group allele and identification of three novel alleles in the Chinese Han population. Vox Sang.

[10] Hakomori S (1991). Immunochemical and molecular genetic basis of the histo-blood group ABO(H) and related antigen system. Baillieres Clin Haematol.

[11] Hosoi E (2008). Biological and clinical aspects of ABO blood group system. J Med Invest.

[12] Storry JR, Olsson ML (2009). The ABO blood group system revisited: a review and update. Immunohematology.

[13] Ying YL, Hong XZ, Xu XG, Chen S, He J, Zhu FM (2020). Molecular basis of ABO variants including identification of 16 novel ABO subgroup alleles in Chinese han population. Transfus Med Hemother.

[14] Hong X, Ying Y, Xu X, Liu Y, Chen ZM, Lan XF (2013). A dispermic chimera was identified in a healthy man with mixed field agglutination reaction in ABO blood grouping and mosaic 46, XY/46, XX karyotype. Transfus Apher Sci.

[15] Storry JR, Castilho L, Daniels G, Arratty G, Francis CL, Moulds JM (2011). International Society of Blood Transfusion Working Party on red cell immunogenetics and blood group terminology: Berlin report. Vox Sang.

[16] Cartegni L, Wang J, Zhu Z, Zhang MQ, Krainer AR (2003). ESEfinder: a web resource to identify exonic splicing enhancers. Nucleic Acids Res.

[17] Desmet FO, Hamroun D, Lalande M, Collod-Béroud G, Claustres M, Béroud C (2009). Human Splicing Finder: an online bioinformatics tool to predict splicing signals. Nucleic Acids Res.

[18] Pertea M, Lin X, Salzberg SL (2001). GeneSplicer: a new computational method for splice site prediction. Nucleic Acids Res.

[19] Reese MG, Eeckman FH, Kulp D, Haussler D (1997). Improved splice site detection in Genie. J Comput Biol.

[20] Brunak S, Engelbrecht J, Knudsen S (1991). Prediction of human mRNA donor and acceptor sites from the DNA sequence. J Mol Biol.

[21] Ryu JS, Lee HY, Cho EH, Yoon KA, Kim MK, Joo J (2020). Exon splicing analysis of intronic variants in multigene cancer panel testing for hereditary breast/ovarian cancer. Cancer Sci.

[22] Seltsam A, Wagner FF, Grüger D, Gupta CD, Bade-Doeding C, Blasczyk R (2007). Weak blood group B phenotypes may be caused by variations in the CCAAT-binding factor/NF-Y enhancer region of the ABO gene. Transfusion.

[23] Sano R, Nakajima T, Takahashi K, Kubo R, Kominato Y, Tsukada J (2012). Expression of ABO blood-group genes is dependent upon an erythroid cell-specific regulatory element that is deleted in persons with the B(m) phenotype. Blood.

[24] Wu PC, Lin YH, Tsai LF, Chen MH, Chen PL, Pai SC (2018). ABO genotyping with next-generation sequencing to resolve heterogeneity in donors with serology discrepancies. Transfusion.

[25] Niwa R, Nakayama T, Ishii H, Fujita E, Ishiyama K, Matsuo T (2016). Identification of a novel missense mutation (563G>a) in the ABO gene associated with a Bel phenotype. Transfusion.

[26] Cai X, Jin S, Liu X, Fan L, Lu Q, Wang J (2013). Molecular genetic analysis of ABO blood group variations reveals 29 novel ABO subgroup alleles. Transfusion.

[27] Ogasawara K, Yabe R, Uchikawa M, Nakata K, Watanabe J, Takahashi Y (2001). Recombination and gene conversion-like events may contribute to ABO gene diversity causing various phenotypes. Immunogenetics.

[28] Yamamoto F, Cid E, Yamamoto M, Blancher A (2012). ABO research in the modern era of genomics. Transfus Med Rev.

[29] Oda A, Isa K, Ogasawara K, Kameyama K, Okuda K, Hirashima M (2015). A novel mutation of the GATA site in the erythroid cell-specific regulatory element of the ABO gene in a blood donor with the Am B phenotype. Vox Sang.

[30] Kronstein-Wiedemann R, Nowakowska P, Milanov P, Knut Gubbe K, Seifried E, Bugert P (2020). Regulation of ABO blood group antigen expression by miR-331-3p and miR-1908-5p during hematopoietic stem cell differentiation. Stem Cells.

[31] Chen DP, Tseng CP, Wang WT, Sun CF (2012). Genetic and mechanistic evaluation for the mixed-field agglutination in B3 blood type with IVS3+5G>A ABO gene mutation. PLoS ONE.

[32] Chen DP, Sun CF, Ning HC, Peng CT, Wang WT, Tseng CP (2015). Genetic and mechanistic evaluation for the weak A phenotype in Ael blood type with IVS6 + 5G>A ABO gene mutation. Vox Sang.

[33] Ying Y, Hong X, Xu X, Liu Y, Lan X, Ma K (2013). Serological characteristic and molecular basis of A2 subgroup in the Chinese population. Transfus Apher Sci.

[34] Kominato Y, Hata Y, Takizawa H, Matsumoto K, Yasui K, Tsukada JI (2002). Alternative promoter identified between a hypermethylated upstream region of repetitive elements and a CpG island in human ABO histo-blood group genes. J Biol Chem.

[35] Ying YL, Tao SD, He YM, Xu XG, Zhu FM, Lv HJ (2011). Molecular mechanism of an individual with weaken B phenotype in ABO blood group. Chin J Med Genet.

[36] Matzhold EM, Drexler C, Wagner A, Bernecker C, Ariane Pessentheiner A, Bogner-Strauß JG (2020). A 24-base pair deletion in the ABO gene causes a hereditary splice site defect: a novel mechanism underlying ABO blood group O. Transfusion.

[37] Cai X, Qian C, Wu W, Lei H, Ding QL, Zou W (2017). An exonic missense mutation c.28G>A is associated with weak B blood group by affecting RNA splicing of the ABO gene. Transfusion.

[38] Hwang DY, Hung CC, Riepe FG, Auchus RJ, Kulle AE, Holterhus PM (2011). CYP17A1 intron mutation causing cryptic splicing in 17α-hydroxylase deficiency. PLoS ONE.

[39] Keren H, Lev-Maor G, Ast G (2010). Alternative splicing and evolution: diversification, exon definition and function. Nat Rev Genet.

