# Supplementary material for: Six splice site variations, three of them novel, in the ABO gene occurring in nine individuals with ABO subtypes
Source: J Transl Med. 2021 Nov 22;19:470. doi: 10.1186/s12967-021-03141-5 (PMC8607603; doi:10.1186/s12967-021-03141-5)
Supplement: Supplementary file 1 — Additional file 1: Figure S1. The heterozygous sequence of the splicing sites in the individuals with ABO subtypes. Arrows indicated the heterozygous variations in ABO gene. Figure S2. The variation sequences of the splicing sites after haploid analysis in the ABO gene. Arrows indicated the variation position. [file 12967_2021_3141_MOESM1_ESM.docx]

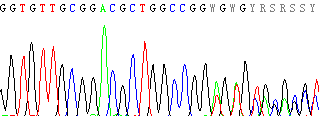

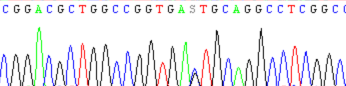


c.28+5G/CG/A

c.28+1_2GT/del

c.28+1


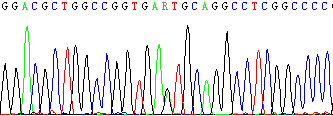

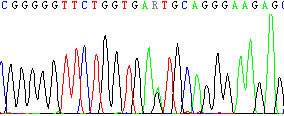


c.28+5G/A

c.155+5G/A


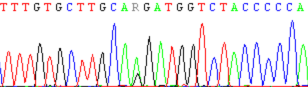

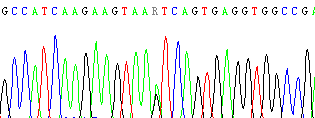


c.374+5G/A

c.204-1G/A

Figure S1 The heterozygous sequence of the splicing sites in the individuals with ABO subtypes. Arrows indicated the heterozygous variations in ABO gene.


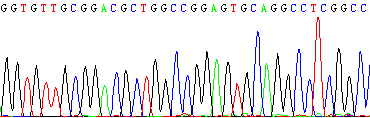

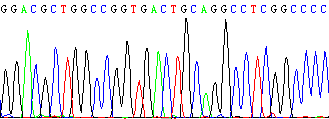


c.28+1_2del

c.28+5C


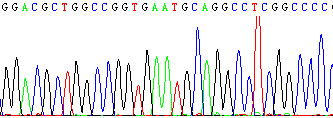

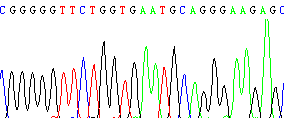


c.28+5A

c.155+5A


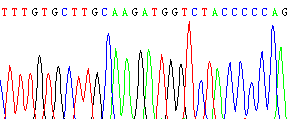

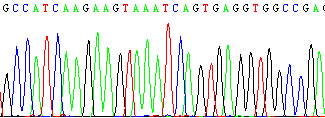

Figure S2 The variation sequences of the splicing sites after haploid analysis and the schematic diagram of splicing site variations in the *ABO* gene. Arrows indicated the variation position.

c.204-1A

c.374+5A
