# Supplementary material for: Six splice site variations, three of them novel, in the ABO gene occurring in nine individuals with ABO subtypes
Source: J Transl Med. 2021 Nov 22;19:470. doi: 10.1186/s12967-021-03141-5 (PMC8607603; doi:10.1186/s12967-021-03141-5)
Supplement: Supplementary file 2 — Additional file 1: Table S1. Nucleotide polymorphism of the ABO gene in specimens ID number3 to 8 by the NGS method. [file 12967_2021_3141_MOESM2_ESM.docx]

Supplement table 1 Nucleotide polymorphism of the *ABO* gene in specimens ID number3 to 8 by the NGS method

| Location* | specimens | | | | | |
| --- | --- | --- | --- | --- | --- | --- |
|  | 3(c.28+5G>C) | 4(c.155+5G>A) | 5(c.155+5G>A) | 6(c.155+5G>A) | 7(c.204-1G>A) | 8(c.374+5G>A) |
| c.28+5 | G/C | G/G | G/G | G/G | G/G | G/G |
| c.28+94 | T/C | C/C | T/C | T/C | T/C | T/T |
| c.28+112 | G/A | A/A | G/A | G/A | G/A | A/A |
| c.28+175 | C/T | C/C | C/C | C/C | C/T | C/T |
| c.28+748 | C/C | C/C | T/C | T/C | C/C | T/C |
| c.28+856 | C/T | C/C | C/C | C/C | C/T | C/T |
| c.28+997 | C/T | C/C | C/C | C/C | C/T | C/T |
| c.28+1078 | G/A | G/A | G/G | G/G | G/A | G/A |
| c.28+1179 | C/C | C/C | T/C | T/C | C/C | T/C |
| c.28+1349 | G/A | G/A | G/G | G/G | G/A | G/A |
| c.28+1428 | G/A | G/A | G/A | G/A | G/A | G/G |
| c.28+1480 | T/A | T/A | T/T | T/T | T/A | T/A |
| c.28+1481_1482 | -/insT | -/insT | -/- | -/- | -/insT | -/insT |
| c.28+1929_1930 | GG/CA | GG/CA | GG/GG | GG/GG | GG/CA | GG/CA |
| c.28+2168 | C/T | C/T | C/C | C/C | C/T | C/T |
| c.28+2209 | G/C | G/C | G/G | G/G | G/C | G/C |
| c.28+2346 | A/G | A/G | A/A | A/A | A/G | A/G |
| c.28+2542 | G/A | G/A | G/G | G/G | G/A | G/A |
| c.28+2577 | C/T | C/T | C/C | C/C | C/T | C/T |
| c.28+2754 | C/A | C/A | C/C | C/C | C/A | C/A |
| c.28+2875 | A/G | A/G | A/A | A/A | A/G | A/G |
| c.28+3024 | C/T | C/T | C/C | C/C | C/T | C/T |
| c.28+3282 | C/C | C/T | C/C | C/C | C/C | C/C |
| c.28+3417 | A/G | A/G | A/A | A/A | A/G | A/G |
| c.28+3565 | G/G | G/A | G/G | G/G | G/G | G/G |
| c.28+3657 | C/A | C/C | C/C | C/C | C/A | C/A |
| c.28+3837 | G/A | G/G | G/G | G/G | G/A | G/A |
| c.28+3913 | T/A | T/A | T/T | T/T | T/A | T/A |
| c.28+3980 | G/G | G/G | A/G | A/G | G/G | A/G |
| c.28+4131 | C/T | C/C | C/C | C/C | C/T | C/T |
| c.28+4143_4144 | -/insGTCTT | -/insGTCTT | -/- | -/- | -/insGTCTT | -/insGTCTT |
| c.28+4161 | A/G | A/G | A/A | A/A | A/G | A/G |
| c.28+4282 | A/G | A/G | A/G | A/G | A/G | A/A |
| c.28+4365 | A/T | A/T | A/A | A/A | A/T | A/T |
| c.28+4515 | C/A | C/A | C/C | C/C | C/A | C/A |
| c.28+4524 | A/del | A/del | A/A | A/A | A/del | A/del |
| c.28+4547 | T/C | T/C | T/T | T/T | T/C | T/C |
| c.28+4577_4578 | -/insAATATTTATTAATTTCCCTTCT | -/insAATATTTATTAATTTCCCTTCT | -/- | -/- | -/insAATATTTATTAATTTCCCTTCT | -/insAATATTTATTAATTTCCCTTCT |
| c.28+4597 | C/T | C/T | C/C | C/C | C/T | C/T |
| c.28+4664 | T/del | T/del | T/T | T/T | T/del | T/del |
| c.28+4781 | C/C | C/C | C/C | C/T | C/C | C/C |
| c.28+5088 | A/T | T/T | A/A | A/A | A/T | A/T |
| c.28+5101 | C/T | T/T | C/C | C/C | C/T | C/T |
| c.28+5334 | A/G | A/G | A/A | A/A | A/G | A/G |
| c.28+5456 | A/G | A/G | A/A | A/A | A/G | A/G |
| c.28+5580 | T/C | T/C | T/T | T/T | T/C | T/C |
| c.28+5614 | T/C | T/C | T/T | T/T | T/C | T/C |
| c.28+5701 | A/G | A/G | A/A | A/A | A/G | A/G |
| c.28+5885 | C/A | C/C | C/C | C/C | C/A | C/A |
| c.28+5948 | C/C | C/T | C/C | C/C | C/C | C/C |
| c.28+5981 | C/A | C/C | C/C | C/C | C/A | C/A |
| c.28+6040 | T/C | T/T | T/T | T/T | T/C | T/C |
| c.28+6120 | T/C | T/C | T/C | T/C | T/C | T/T |
| c.28+6147 | G/A | G/A | G/G | G/G | G/A | G/A |
| c.28+6265_6266 | TG/CA | TG/CA | TG/TG | TG/TG | TG/CA | TG/CA |
| c.28+6277 | C/T | C/T | C/C | C/C | C/T | C/T |
| c.28+6290 | T/A | T/A | T/T | T/T | T/A | T/A |
| c.28+6464 | T/C | T/T | T/T | T/T | T/C | T/C |
| c.29-6479 | T/T | T/A | T/T | T/T | T/T | T/T |
| c.29-5862 | C/T | C/T | C/C | C/C | C/T | C/T |
| c.29-5792 | A/G | A/G | A/A | A/A | A/G | A/G |
| c.29-5632 | T/C | T/C | T/T | T/T | T/C | T/C |
| c.29-5540_5539 | GC/AT | GC/AT | GC/GC | GC/GC | GC/AT | GC/AT |
| c.29-5419 | A/T | A/T | A/A | A/A | A/T | A/T |
| c.29-4882 | T/G | T/T | T/T | T/T | T/G | T/G |
| c.29-4774 | T/C | T/C | T/T | T/T | T/C | T/C |
| c.29-4732 | T/G | T/G | T/G | T/G | T/G | T/T |
| c.29-4636 | C/A | C/A | C/C | C/C | C/A | C/A |
| c.29-4622 | G/T | G/T | G/G | G/G | G/T | G/T |
| c.29-4604 | G/A | G/A | G/A | G/A | G/A | G/G |
| c.29-4419 | G/C | G/G | G/G | G/G | G/C | G/C |
| c.29-4288 | G/G | G/G | A/G | A/G | G/G | A/G |
| c.29-3924 | C/T | C/T | C/T | C/T | C/T | C/C |
| c.29-3556 | A/G | A/A | A/A | A/A | A/G | A/G |
| c.29-2934 | G/A | G/G | G/G | G/G | G/A | G/A |
| c.29-2883 | A/G | A/A | A/A | A/A | A/G | A/G |
| c.29-2337_2336 | InsGGCAGTT/insGGCAGTT | InsGGCAGTT/insGGCAGTT | -/insGGCAGTT | -/insGGCAGTT | InsGGCAGTT/insGGCAGTT | -/insGGCAGTT |
| c.29-2183 | A/A | A/G | A/A | A/A | A/G | A/A |
| c.29-2046 | G/A | G/G | G/G | G/G | G/A | G/A |
| c.29-1949 | G/G | G/G | G/G | G/A | G/G | G/G |
| c.29-1726 | G/G | G/A | G/G | G/G | G/A | G/G |
| c.29-1694 | T/G | T/G | T/T | T/T | G/G | T/G |
| c.29-1205 | A/A | A/G | A/A | A/A | A/G | A/A |
| c.29-1053_1037 | AACAACAACAAAACAAC/del | AACAACAACAAAACAAC/del | AACAACAACAAAACAAC/AACAACAACAAAACAAC | AACAACAACAAAACAAC/AACAACAACAAAACAAC | AACAACAACAAAACAAC/del | AACAACAACAAAACAAC/AACAACAACAAAACAAC |
| c.29-746 | T/T | T/C | T/T | T/T | T/T | T/T |
| c.29-658 | G/G | G/A | G/G | G/G | G/G | G/G |
| c.29-554 | A/C | A/A | A/A | A/A | A/C | A/C |
| c.29-286 | A/C | A/A | A/A | A/A | A/C | A/C |
| c.29-86 | G/A | G/A | G/A | G/A | G/A | G/G |
| c.98+362 | C/C | C/T | C/C | C/C | C/C | C/C |
| c.99-356 | C/C | C/G | C/C | C/C | C/C | C/C |
| c.99-329 | T/C | T/C | T/T | T/T | T/C | T/C |
| c.99-288 | C/T | C/T | C/C | C/C | C/T | C/T |
| c.99-186 | C/C | C/A | C/C | C/C | C/C | C/C |
| c.106G>T | G/T | G/G | G/G | G/G | G/T | G/T |
| c.155+5 | G/G | G/A | G/A | G/A | G/G | G/G |
| c.155+138 | C/T | C/C | C/C | C/C | C/C | C/C |
| c.155+205 | C/T | C/C | C/C | C/C | C/T | C/T |
| c.155+479 | C/T | C/C | C/C | C/C | C/T | C/T |
| c.155+525 | A/T | A/A | A/A | A/A | A/T | A/T |
| c.155+575 | C/C | C/T | C/C | C/C | C/C | C/C |
| c.155+691 | C/T | C/C | C/C | C/C | C/C | C/C |
| c.156-483 | T/C | T/T | T/T | T/T | T/C | T/C |
| c.156-389 | A/G | A/A | A/A | A/A | A/G | A/G |
| c.156-208 | C/T | C/C | C/C | C/C | C/T | C/T |
| c.156-174 | T/C | T/T | T/T | T/T | T/C | T/C |
| c.156-95 | C/T | C/C | C/C | C/C | C/T | C/T |
| c.188_189 | GC/AT | GC/GC | GC/GC | GC/GC | GC/AT | GC/AT |
| c.203+28 | G/C | G/G | G/G | G/G | G/C | G/C |
| c.203+72_73 | -/insGTGTGGACAGAAG | -/- | -/- | -/- | -/insGTGTGGACAGAAG | -/insGTGTGGACAGAAG |
| c.203+102 | C/C | C/A | C/C | C/C | C/C | C/C |
| c.203+114 | C/T | C/C | C/C | C/C | C/T | C/T |
| c.203+163 | G/A | G/G | G/G | G/G | G/A | G/A |
| c.203+215_216 | AA/GC | AA/AA | AA/AA | AA/AA | AA/GC | AA/GC |
| c.203+346 | T/G | T/T | T/T | T/T | T/G | T/G |
| c.203+413 | C/C | C/C | C/C | C/T | C/C | C/C |
| c.203+738 | T/G | T/T | T/G | T/G | T/G | T/G |
| c.204-545 | C/C | C/C | T/T | T/T | C/C | T/T |
| c.204-511 | C/T | C/C | C/C | C/C | C/T | C/C |
| c.204-220 | G/A | G/G | G/G | G/G | G/A | G/A |
| c.204-191 | T/C | T/T | T/T | T/T | T/C | T/C |
| c.204-176 | T/G | T/T | T/T | T/T | T/G | T/G |
| c.204-61_62 | -/insC | -/- | -/- | -/- | -/insC | -/insC |
| c.204-9 | T/T | T/C | T/T | T/C | T/T | T/T |
| c.204-1 | G/G | G/G | G/G | G/G | G/A | G/G |
| c.220 | C/T | C/C | C/C | C/C | C/T | C/T |
| c.239+103_104 | -/insCCC | -/- | -/- | -/- | -/insCCC | -/insCCC |
| c.240-249 | C/T | C/C | C/C | C/C | C/T | C/T |
| c.240-219 | G/G | G/A | G/G | G/G | G/G | G/G |
| c.240-105 | C/A | C/C | C/C | C/C | C/A | C/A |
| c.240-28 | G/A | G/G | G/G | G/G | G/A | G/A |
| c.240-25 | A/G | A/G | A/G | A/G | A/G | A/A |
| c.261 | G/del | G/del | G/G | G/G | G/del | G/del |
| c.297 | G/G | A/G | A/G | A/G | G/G | A/G |
| c.374+5 | G/G | G/G | G/G | G/G | G/G | G/A |
| c.374+42 | G/T | G/T | G/T | G/T | G/T | G/G |
| c.374+89 | T/A | T/T | T/T | T/T | T/A | T/A |
| c.374+163 | C/C | C/T | C/T | C/T | C/C | C/T |
| c.374+179 | T/C | T/C | T/C | T/C | T/C | C/C |
| c.374+188 | G/A | G/G | G/G | G/G | G/A | G/A |
| c.374+226 | C/T | C/C | C/C | C/C | C/T | C/T |
| c.374+235 | C/G | C/C | C/C | C/C | C/G | C/G |
| c.374+271 | A/G | A/G | A/G | A/A | A/G | A/A |
| c.374+280 | C/T | C/T | C/T | C/T | C/T | C/C |
| c.374+446 | G/G | A/G | A/G | A/A | G/G | A/G |
| c.374+493 | T/C | T/T | T/T | T/T | T/C | T/T |
| c.375-425 | A/G | A/G | A/G | A/G | A/G | A/A |
| c.375-336 | G/A | G/G | G/G | G/G | G/A | G/G |
| c.375-269 | G/G | G/A | G/G | G/G | G/G | G/G |
| c.375-267 | G/G | A/G | A/G | A/G | G/G | A/G |
| c.375-162 | G/G | A/G | A/G | A/G | G/G | A/G |
| c.375-152 | G/A | G/A | G/A | G/A | G/A | G/G |
| c.375-103 | G/G | A/G | A/G | A/G | G/G | A/G |
| c.375-42 | A/G | A/A | A/A | A/A | A/G | A/G |
| c.375-40 | G/A | G/G | G/G | G/G | G/A | G/A |
| c.467 | C/C | C/C | C/T | C/T | C/C | C/T |
| c.526 | C/G | C/G | C/G | C/G | C/G | C/C |
| c.646 | T/A | T/T | T/T | T/T | T/A | T/A |
| c.657 | C/T | C/T | C/T | C/T | C/T | C/C |
| c.681 | G/A | G/G | G/G | G/G | G/A | G/A |
| c.703 | G/A | G/A | G/A | G/A | G/A | G/G |
| c.771 | C/T | C/C | C/C | C/C | C/T | C/T |
| c.796 | C/A | C/A | C/A | C/A | C/A | C/C |
| c.803 | G/C | G/C | G/C | G/C | G/C | G/G |
| c.829 | G/A | G/G | G/G | G/G | G/A | G/A |
| c.930 | G/A | G/A | G/A | G/A | G/A | G/G |

*The position of intron sequence was according to sequence of the NG_006669.2. c.28+94 indicates to the 94th base (intron sequence) starting from position 28 of cDNA.
